# Water source and diarrhoeal disease risk in children under 5 years old in Cambodia: a prospective diary based study

**DOI:** 10.1186/1471-2458-13-1145

**Published:** 2013-12-09

**Authors:** Paul R Hunter, Helen Risebro, Marie Yen, Hélène Lefebvre, Chay Lo, Philippe Hartemann, Christophe Longuet, François Jaquenoud

**Affiliations:** 1 Norwich School of Medicine, University of East Anglia, Norwich NR4 7TJ, UK; 2 Department of Environmental Health, Tshwane University of Technology, Pretoria, South Africa; 3 1001 fontaines pour demain, Caluire et Cuire, France; 4 Teuk Saat 1001, Phnom Penh, Cambodia; 5 Département Environnement et Santé Publique, Faculté de médecine de Nancy - Université de Lorraine, Nancy, France; 6 Fondation Mérieux, Lyon, France

**Keywords:** Water, Diarrhoea, Latrines, Cambodia, Child health, Bottled water, Disinfection

## Abstract

**Background:**

Despite claims that the Millennium Development Goals (MDG) targets on access to safe drinking water have been met, many 100 s of millions of people still have no access. The challenge remains how to provide these people and especially young children with safe drinking water.

**Method:**

We report a longitudinal study designed to assess the effectiveness of an intervention based on provided treated drinking water in containers on self-reported diarrhoea in children. The intervention was “1001 fontaines pour demain” (1001 F) is a non-governmental not for profit organization (created in 2004 and based in Caluire, France) that helps local entrepreneurs treat package, and sell safe drinking water. Cases and controls were chosen at village and household level by propensity score matching Participants were visited twice a month over six months and asked to complete a diarrhoea health diary.

**Results:**

In total 4275 follow-up visits were completed on 376 participants from 309 homes. Diarrhoea was reported in 20.4% of children on each visit, equating to an incidence rate estimate of 5.32 episodes per child per year (95% confidence interval = 4.97 to 5.69). Compared to those drinking 1001 F water, children drinking surface water were 33% (95% CI -1 to 17%), those drinking protected ground water were 62% (95% CI 19 to 120%) and those drinking other bottled water 57% (95% CI 15 to 114%) more likely to report diarrhoea. Children drinking harvested rainwater had similar rates of diarrhoea to Children drinking 1001 F water.

**Conclusion:**

Our study suggests that 1001 F water provides a safer alternative to groundwater or surface water. Furthermore, our study raises serious concerns about the validity of assuming protected groundwater to be safe water for the purposes of assessing the MDG targets. By contrast our study provides addition evidence of the relative safety of rainwater harvesting.

## Background

Diarrhoeal disease is one of the most important causes of disease burden and mortality in children under 5 years old [1]. Most of this disease burden falls on those children growing up in the world’s poorest countries and is largely associated with inadequate drinking water and sanitation [2]. In recognition of this, improved access to safe drinking water and sanitation has been one of the key aspects of the millennium development goals (MDG) [3]. Although recent statements from the United Nations and World Health Organization have claimed that the MDG on water access has been met ahead of target [4], there have been cogent arguments that these claims are exaggerated [5]. In any event, even if the MDG targets have been met in full there still remains many 100 s of millions of people without access to sustainable safe water supplies.

In this regard the issue of whether improved water quantity (access) or quality is most important for protecting child health is central to policy on improving water supplies. The quantity versus quality debate has continued since the late 1980s [6]. However, at least in the view of the authors of this article, there is sufficient evidence to support the conclusion that both quality and quantity are important [6-9]. Once one accepts the importance of good quality drinking water, then the question becomes how can people access a sufficient, reliable and sustainable source of safe drinking water? Whilst the preferred option must always be properly managed community mains drinking water treatment and distribution networks, this is not possible for many communities because of either financial or geographical considerations. In such communities the options for obtaining safe drinking water are limited to accessing other sources of “improved” drinking water. According to the WHO / UNICEF Joint Monitoring Programme (JMP) for Water Supply and Sanitation “An improved drinking-water source is defined as one that, by nature of its construction or through active intervention, is protected from outside contamination, in particular from contamination with faecal matter” (WHO/UNICEF Joint Monitoring Programme (JMP) for Water Supply and Sanitation http://www.wssinfo.org/definitions-methods/introduction/). Under JMP definitions, improved drinking water sources include piped water into dwelling or yard, public taps or standpipes, tubewells or boreholes, protected dug wells, protected springs and rainwater sources. Unimproved water sources include unprotected springs, unprotected dug wells, carts with small tanks or drums, tanker-trucks, surface waters and bottled waters.

For people without access to improved drinking water supplies the options are much more limited. Household Water Treatment is being heavily promoted in many parts of the world despite the generally poor evidence that these technologies are effective in reducing self-reported diarrhoeal disease. In particular, double blinded trials of household chlorination have not found any impact on diarrhoeal disease [10]. The evidence in favour of other technologies such as solar disinfection (SODIS) is also weak with independent studies suggesting poor compliance and little if any strong evidence of impact on health with support only coming from unblinded trials [11]. By contrast the evidence in favour of ceramic filters being effective in reducing self-reported diarrhoea is somewhat stronger [11]. The reasons for the lack of effect of Household Water Treatment probably includes poor effectiveness against some pathogens (especially chlorination and *Cryptosporidium*) and poor compliance [12,13].

In the JMP classification, bottled/packaged waters are deemed to be an unimproved water supply and are, therefore, considered to be at risk of contamination (WHO/UNICEF Joint Monitoring Programme (JMP) for Water Supply and Sanitation http://www.wssinfo.org/definitions-methods/introduction/). This classification is almost certainly correct as the quality of bottled water can be variable [14-17]. However, the question remains whether or not bottle water can be produced to an acceptable quality that would be associated with improved health impacts at a price that would make it an economically acceptable choice for the primary source of drinking water. In this paper we report a longitudinal study of diarrhoeal disease in children under five in communities where a particular type of packaged water, 1001Fontaines, was sold and in communities where this water was not available.

## Methods

We undertook a prospective longitudinal survey of parent-reported diarrhoeal disease in children under 5 years old in rural communities in Cambodia. Participating households were visited every 2 weeks and a short questionnaire administered to determine, amongst other things, the incidence of acute diarrhoea in children and drinking water sources.

### The 1001 F model

“1001 fontaines pour demain” (1001 F) is a non-governmental not for profit organization (created in 2004 and based in Caluire, France) whose primary goal is to improve access to safe drinking water. This initiative is specifically orientated towards small rural communities, which generally fall outside of the remit of water access projects. Between 2005 and 2011, 1001 F has implemented several pilot projects in Cambodia, enabling by the end of 2012, approximately 65,000 people in 58 villages to have access to safe water. 1001 F does this by supporting water entrepreneurs in communities to build water treatment and distribution businesses.

The basic business model is to support local entrepreneurs to build a water treatment system, based on filtration and ultraviolet disinfection of source water with bottling in cleaned and disinfected 20 L containers for subsequent distribution and sale. A continuing quality assurance programme is implemented with support from 1001 F technical staff. The entrepreneurs are supported and trained over an initial apprenticeship year by the end of which the business is financially self-sustaining. After that first year ongoing support is provided by a team of technicians which gives technical assistance and carries out quality control tasks for 50 to 60 sites. Water treatment plants are powered by solar energy. The cost to consumers is less than US$0.01 per litre.

Funding for 1001 F comes mainly from private donors (private companies, foundations), although it has also received financial support from the French Embassies in Cambodia and Madagascar. A video showing more information can be found at the following link: http://fr.youtube.com/watch?v=8bykbVECVrE.

### Participant selection and recruitment

Participant selection was a two phase process; firstly selection of villages and secondly selection of households within villages. For village selection, data concerning 56 villages were obtained from The National Institute of Statistics, Ministry of Planning, Royal Government of Cambodia (http://www.nis.gov.kh/index.php/online-statistics/resultonline). This data included the number of homes in the village, the population, % male, % children <14, % children <5, % of population >25 years with no, some, completed primary and completed secondary education, % migrants, % with access to improved water supplies, % with no toilet facility, % adult female literacy and % employed in primary, secondary and tertiary sectors. Of the 56 villages, 27 had access to 1001 F water. With this data, propensity scores (the probability of being an intervention village) were calculated using logistic regression analysis as has been done in previous studies [18,19]. The most closely matched 1001 F and control village pair was then chosen for inclusion, followed by the next most closely matched, etc.

All households within chosen villages were then visited and a recruitment questionnaire administered covering a range of demographic, socio-economic and environmental variables. This questionnaire also asked about whether or not 1001 F water was the primary source of drinking water. At this first visit, respondents were asked whether or not they would be prepared to participate in the prospective study. Using just the data from 1001 F villages a propensity score model was derived for whether or not the household consistently used 1001 F water using binary logistic regression. The same model was then applied to households in control village in order to identify those households who would most likely purchase 1001 F water should this become available. Frequency matching was then done across ranges of propensity scores between households using 1001 F water and those households with similar scores in control villages.

### Data collection

The first visits in the prospective part of the study were done on the 8^th^ December 2011. Each respondent was given a diary in which to record diarrhoea and or vomiting every day for each child. Households were then visited every two weeks for a total of 12 visits. At each visit the interviewer read the diarrhoea diary and asked about whether or not the drinking water source had changed since the previous visit and whether or not the child had been away from home.

The case definition for an episode of diarrhoea was 3 or more episodes of diarrhoea in a 24 period or any number plus vomiting.

### Data analysis and sample size calculation

All analyses were done with SPSS version 18. To determine incidence rate ratios we used Generalized Estimating Equations for negative binomial family and log link. In order to account for repeat sampling of the same individual and possible within group correlation, person, household and village level were specified as subject variables. An autoregressive (1) working matrix was chosen because of a degree of autocorrelation between visits. As well as using propensity score matching to match people in villages without access to 1001 F to users in villages with such access, a range of potential confounding variables with tested in single predictor analyses. Those confounders that were significantly associated with diarrhoea at the p < 0.2 level were included in a multiple predictor model. The least significant potential confounding variable was removed and the model re-run until all variables were significant at the p < 0.2 level.

### Ethical approval

This study was reviewed and approved by the ethical review committee of the Faculty of Health, University of East Anglia and National Ethics Committee for Health Research of Cambodia. Informed consent was obtained from the parents or guardians of all children participating in this study.

## Results

For several of the originally chosen intervention villages, only a small number of households using 1001 F water and with children living at home were recruited. Where this was the case the intervention village with the closest propensity score and not already included was also added to the study. Any recruited volunteers from the original village were still included in the study. Eventually households from 25 villages were included in the prospective study. Of these 25 villages, 15 were 1001 F villages and 10 control villages.

**Figure 1:**
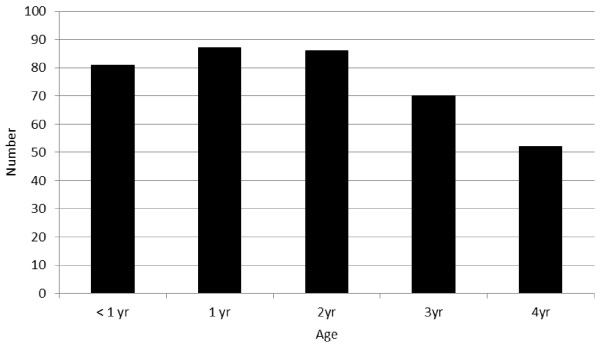
Age distribution of participants on recruitment into the study.

In total 4275 follow-up visits were completed on 376 participants from 309 homes. Of these 376 participants, 340 (90.4%) were included on all 12 occasions. Of the 36 that were not included in all visits 12 were recruited after the study start because they were born into families already recruited, leaving 24 (6.4%) who either died, moved away or decided not to participate further. Key demographic characteristics of the recruited population are shown in Table 1 for the intervention and non-intervention recruits. Figure 1 shows the distribution of ages on recruitment to the study. From the initial analysis it would appear that the populations using 1001 F water and not were very similar on all key variables including propensity score, household size, age at recruitment, anthropometry, family income and mother’s education (Table 1).

**Table 1:** Demographics of the recruited populations in intervention and non-intervention communities

**Variable**	**1001 F user**	**N**	**Mean**	**Std Dev**	**P**
Propensity score	N	165	0.46	0.21	0.707
	Y	211	0.47	0.27	
Number of adults in home	N	165	2.70	1.16	0.653
	Y	211	2.76	1.20	
Number of children in home	N	165	2.37	1.33	0.177
	Y	211	2.76	1.14	
Age at recruitment	N	165	2.18	1.31	0.785
	Y	211	2.21	1.29	
Height/cm at recruitment	N	165	78.0	13.7	0.285
	Y	209	79.4	12.4	
Weight/Kg at recruitment	N	165	10.0	3.1	0.147
	Y	209	10.5	2.8	
			Proportion		
Proportion male	N	165	0.47		0.603
	Y	211	0.50		
Proportion mothers with secondary or higher education	N	165	0.36		0.966
	Y	211	0.36		
			Median		
Monthly income	N	165	US$101-150		0.225
	Y	211	US$101-150		

One of the key findings was that households’ choice of water often varied from one visit to the next. Indeed 98 (32%) households reported changing their main drinking water source from the previous visit at least once. It can be seen from Figure 1 that the main change was a reduction in use of 1001 F water an increase in use of rainwater during April and May (the rainy season). Also of note was an increased use of bottled water other than 1001 F water. Most times this water was described as “Pure water” which was a bottled water of unknown provenance and uncertain source/treatment.

**Figure 2:**
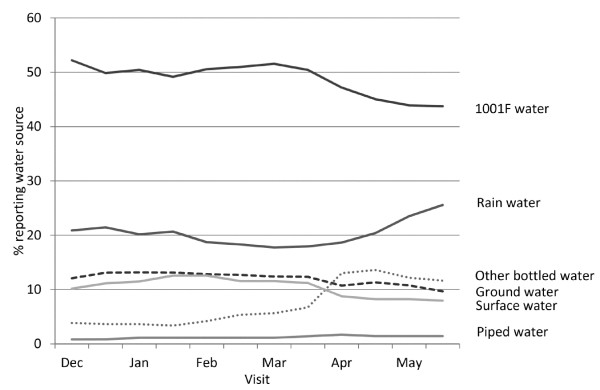
Percentage of households (both intervention and control) reporting main source of drinking water at each visit.

Overall diarrhoea was reported in 20.4% of children on each visit. This equates to an incidence rate estimate of 5.32 episodes per child per year (95% confidence interval = 4.97 to 5.69). The risk factors associated with disease risk in a single variable analysis are listed in Table 2. Figure 2 shows the crude estimates of diarrhoea in people consuming water from different water sources.

**Table 2:** Generalized estimating equation single predictor variable analysis of diarrhoeal disease risk in children under 5 years old accounting for repeat measures within individual and possible clustering in home and village

**Variable**		**IRR**	**L95% CI**	**U95% CI**	**P**
Gender	F	1			0.275
	M	0.882	0.703	1.105	
Age	/y	0.819	0.748	0.879	<0.0001
Visit number	1	1			4.6 × 10^-16^
	2	2.116	1.649	2.716	
	3	1.962	1.489	2.586	
	4	1.689	1.288	2.216	
	5	1.604	1.205	2.134	
	6	1.615	1.21	2.155	
	7	1.255	0.924	1.705	
	8	1.266	0.926	1.732	
	9	1.043	0.747	1.458	
	10	0.863	0.607	1.226	
	11	1.167	0.851	1.601	
	12	0.900	0.634	1.279	
Child spent night away from home in previous 2 weeks	N	1			0.004
	Y	1.313	1.094	1.577	
Any child in family spent night away from home in previous 2 weeks	N	1			0.001
	Y	1.340	1.125	1.596	
Another case in house	N	1			<0.0001
	Y	2.167	1.852	2.536	
Mother’s education	Primary or less	1			
	Secondary or higher	0.840	0.663	1.064	0.149
Household monthly income	/income band	0.899	0.833	0.971	0.007
Sanitation	Open defecation	1			0.021
	Use latrine	0.762	0.605	0.960	
Water source	1001 F water	1			0.0001
	Piped supply	0.322	0.096	1.086	
	Rainwater	0.946	0.719	1.244	
	Protected groundwater	1.625	1.198	2.205	
	Other container water	1.602	1.181	2.172	
	Surface water	1.478	1.095	1.993	
	Unprotected groundwater	O.936	0.399	2.193	
Recently changed water source	No	1			0.008
	Yes	1.335	1.079	1.652	
Adults in home	/person	0.942	0.832	1.067	0.345
Children in home	/child	1.036	0.943	1.139	0.462
Propensity score	/1.0	0.690	0.440	1.000	0.106

Table 3 shows the final model after adjustment for the visit number, age, whether or not the child spent a night away from home in the previous two weeks, whether or not people use latrines or open defecation, water source, and whether or not the household recently changed their drinking water source were all associated with risk of illness. Whether or not there was a further case of diarrhoea in the home was not included in the final model as when there were more than one case it was not clear whether they were co-primaries or which was the primary and which the secondary case. Of particular interest for this study was that compared to children drinking 1001 F water, children drinking surface water were 33% more likely to report diarrhoea (95% CI -1 to 17%), those drinking protected ground water were 62% (95% CI 19 to 120%) and those drinking other bottled water 57% (95% CI 15 to 114%) more likely to report diarrhoea. By contrast those using a piped supply were 72% (95% CI 20 to 90%) less likely to report diarrhoea. Also of interest were the findings that those drinking rainwater and unprotected ground water had very similar illness rates to those drinking 1001 F water. However very few people reported drinking from unprotected groundwater sources and the confidence intervals were very wide.

**Table 3:** Final generalized estimating equation model of factors associated with risk of diarrhoea in children under 5 years old accounting for repeat measures within individual and possible clustering in home and village

**Variable**		**IRR**	**L95% CI**	**U95% CI**	**P**
**Age**	**/y**	**0.830**	**0.760**	**0.907**	**0.00004**
Visit Number	1	1			4.4 × 10^-15^
	2	2.062	1.603	2.653	
	3	1.909	1.44	2.529	
	4	1.662	1.259	2.194	
	5	1.592	1.193	2.125	
	6	1.637	1.22	2.196	
	7	1.265	0.928	1.726	
	8	1.274	0.928	1.75	
	9	0.996	0.71	1.398	
	10	0.812	0.568	1.161	
	11	1.158	0.838	1.6	
	12	0.899	0.628	1.286	
Any child spent night away from home in previous 2 weeks	N	1			0.003
	Y	1.289	1.09	1.524	
Household monthly income	/US$50 income band	0.945	0.878	1.018	0.137
Sanitation	Open defecation	1			0.036
	Use latrine	0.782	0.621	0.984	
Water source	1001 F water	1			0.0004
	Piped supply	0.284	0.101	0.8	
	Rainwater	0.998	0.754	1.32	
	Protected groundwater	1.619	1.192	2.199	
	Other container water	1.569	1.149	2.141	
	Surface water	1.326	0.988	1.778	
	Unprotected groundwater	1.041	0.489	2.216	
Recently changed water source	No	1			0.014
	Yes	1.295	1.054	1.591	

One of the findings in this study was the impact of recently changing water source on diarrhoeal risk. If the household source of drinking water had changed since the previous visit then there was an additional risk of diarrhoea of 30% (95% CI 5 to 59%). Although not the primary focus of this study we also found that use of latrines was associated with a 22% (95% CI 2 to 38%) reduction in diarrhoea compared to those using open defecation.

## Discussion

This study was designed to investigate whether purchase of 1001 F water was associated with a reduced risk of diarrhoeal disease in children under five years old. We have shown that 1001 F as a drinking water source is associated with significantly reduced risk compared to many other widely used water sources in rural Cambodia, including surface water, protected groundwater and other bottled water. We would argue that the health benefits of consumption of 1001 F water over the alternatives are because of the design and management of the plants and processes, most importantly the use of UV disinfection that provides a wider kill spectrum against waterborne pathogens over alternatives [20]. However, probably the most important aspect is the initial training of entrepreneurs followed by the continued technical support and external quality control provided. In previous work we have shown that improved training of people managing water distribution in poor communities can be associated with health gains [21].

It should be stated that this was an observational and not an experimental study. As such participants were not blinded to their sources of drinking water and were clearly aware what water source they were using. Furthermore, the choice of whether or not to purchase water is governed by a range of other socio-economic factors. For both these reasons there is the potential for any findings to be affected by a range of biases and confounding [22]. For example, in one study of Household Water Treatment using Solar Disinfection (SODIS), the authors reported that people previously involved with the implementation were likely to over-estimate compliance compared to independent investigators [23].

To minimize the potential for reporting bias, we employed an independent survey company that had no prior involvement with 1001 F, and enumerators were trained on the importance of impartiality and not to suggest the value of one water source over another. To further minimize the potential for recall bias we provided participants with a daily diarrhoea diary so that they would not have to remember episodes of illness until the next visit. Also because this was an observational study rather than a randomised trial it was easier to prevent participants from being aware of our study hypotheses. To overcome the problem of confounding we used a two stage propensity score matching process. This process ensured that we were able to compare people in villages that had access to 1001 F water with people in villages that were as similar as possible. But perhaps more importantly, within those villages without access to 1001 F we were able to choose participants who were as similar as possible to people who were using 1001 F water in villages with access, i.e. people who would probably use 1001 F water if and when it became available. By these processes we have controlled for these sources of bias as best as we were able. However, even a process of propensity score matching is not a guarantee that all potential confounding variables are controlled for.

A particularly important finding was the observation that risk in people when drinking rainwater and 1001 F water was almost identical. Although harvested rainwater is classed as an improved drinking water source, a number of authors have expressed concerns about the safety of rainwater harvesting as a drinking water source based on microbiological examination [24,25]. However, in a recent systematic review and meta-analysis of epidemiological studies of diarrhoea and consumption of rainwater we showed that harvested rainwater was associated with a reduced risk compared to unimproved water sources and had similar risk as improved supplies such as mains water in most studies [26]. Our study provides further evidence for the relative safety of rainwater harvesting.

Of particular concern is the finding that protected groundwater sources were associated with the highest risk of illness. Protected groundwater sources are classified as improved water sources and so are considered to be protected from contamination with faecal matter (WHO/UNICEF Joint Monitoring Programme (JMP) for Water Supply and Sanitation http://www.wssinfo.org/definitions-methods/introduction/). Perhaps this should not be such a great surprise as microbiological studies of such groundwaters often report high rates of *E. coli* indicating faecal contamination [27-29]. Because protected groundwater supplies are considered to be at low risk of contamination, communities reliant on these supplies are deemed to be provided with safe water under the requirements of the Millennium Development Goals (MDG). Many of those people now classed as having access to safe water would be so classed on the basis of access to protected groundwater. Our results cast serious doubt on the safety of protected ground waters and consequently further doubt on the validity of the claim that the MDG target on access to safe drinking water has been met.

Although this study was not primarily concerned with sanitation other than as a potential confounder of water supply on disease risk, we have shown an important impact of latrine use in reducing disease risk. Despite the fact that improved sanitation is widely accepted as being one of the most important public health interventions in recent history [30], there is relatively little firm evidence of its value in development settings [31]. Our study would add important further support for the importance of sanitation improvements.

A final comment about the 1001 F programme is because of the focus on helping local entrepreneurs develop sustainable locally based businesses; this intervention supports economic development in a way that retains funds within the communities themselves. As we have shown previously in a very different setting, education and training around water supply can have an impact beyond water quality and support people in climbing out of poverty [21].

## Conclusions

In conclusion this study has suggested that consumption of 1001 F water is associated with a reduction in acute diarrhoeal disease in young children. We have argued that the care taken in participant selection, the use of diaries rather than relying on memory and using an independent survey company would reduce biases and improve confidence in our conclusions. In addition our study has provided further evidence in support of the safety of rainwater harvesting. However, our work also suggests that protected groundwater sources may not actually provide safe drinking water casting further doubt on claims that the MDG on drinking water has been met.

## Competing interests

PRH was until 2010 a member of the science advisory council of Suez Environment, a water utility. He also received an honorarium to attend a workshop held by Danone Group. Otherwise the authors declare no competing interests.

## Authors’ contributions

PRH, HR, MY, HL, PH, CL and FJ were responsible for study design. MY, HL, CL and FJ were responsible for data collection, PRH undertook the analysis and wrote the first draft of the manuscript, all authors were involved in preparation and approval of the final draft of the manuscript.

## Pre-publication history

The pre-publication history for this paper can be accessed here:

http://www.biomedcentral.com/1471-2458/13/1145/prepub
